# Long-Term follow up after intra-Uterine transfusionS; the LOTUS study

**DOI:** 10.1186/1471-2393-10-77

**Published:** 2010-12-01

**Authors:** Esther P Verduin, Irene TM Lindenburg, Vivianne EHJ Smits-Wintjens, Jeanine MM van Klink, Henk Schonewille, Inge L van Kamp, Dick Oepkes, Frans J Walther, Humphrey HH Kanhai, Ilias IN Doxiadis, Enrico Lopriore, Anneke Brand

**Affiliations:** 1 Division Research, Department of Transfusion Medicine, Sanquin Blood Supply Foundation, Leiden, the Netherlands; 2 Department of ImmunoHematology & Bloodtransfusion, Leiden University Medical Center, Leiden, the Netherlands; 3 Department of Obstetrics, Leiden University Medical Center, Leiden, the Netherlands; 4 Division of Neonatology, Department of Pediatrics, Leiden University Medical Center, Leiden, the Netherlands; 5 Department of Medical Psychology, Leiden University Medical Center, Leiden, the Netherlands

## Abstract

**Background:**

The Leiden University Medical Center (LUMC) is the Dutch national referral centre for pregnancies complicated by haemolytic disease of the fetus and newborn (HDFN) caused by maternal alloimmunization. Yearly, 20-25 affected fetuses with severe anaemia are transfused with intra-uterine blood transfusions (IUT). Mothers of whom their fetus has undergone IUT for HDFN are considered high responders with regard to red blood cell (RBC) antibody formation. Most study groups report high perinatal survival, resulting in a shift in attention towards short- and long-term outcome in surviving children.

**Methods/Design:**

We set up a large long-term observational follow-up study (LOTUS study), in cooperation with the Sanquin Blood Supply Foundation and the LUMC departments of Obstetrics, Neonatology and ImmunoHematology & Bloodtransfusion.

The first part of this study addresses several putative mechanisms associated with blood group alloimmunization in these mothers. The second part of this study determines the incidence of long-term neurodevelopment impairment (NDI) and associated risk factors in children treated with IUT. All women and their life offspring who have been treated with IUT for HDFN in the LUMC from 1987-2008 are invited to participate and after consent, blood or saliva samples are taken. RBC and HLA antigen profile and antibodies are determined by serologic or molecular techniques. Microchimerism populations are tested by real time polymerase chain reaction (RT PCR).

All children are tested for their neurological, cognitive and psychosocial development using standardised tests and questionnaires. The primary outcome is neurodevelopmental impairment (NDI), a composite outcome defined as any of the following: cerebral palsy, cognitive or psychomotor development < 2 standard deviation, bilateral blindness and/or bilateral deafness.

**Discussion:**

The LOTUS study includes the largest cohort of IUT patients ever studied and is the first to investigate post-IUT long-term effects in both mother and child. The results may lead to a change in transfusion policy, in particular future avoidance of certain incompatibilities. Additionally the LOTUS study will provide clinicians and parents better insights in the long-term neurodevelopmental outcome in children with HDFN treated with IUTs, and may improve the quality of antenatal counselling and long-term guidance.

## Background

Alloimmunization is a major transfusion problem and deliberate transfusions may induce multiple red cell, platelet and HLA specific antibodies. Some transfusion recipients seem more susceptible for alloimmunization, but with respect to red cell (RBC) antibodies the mechanisms have hardly been investigated. If a pregnant woman has RBC alloantibodies of the IgG class, which can cross the placenta, this may lead to haemolytic disease of the fetus and newborn (HDFN). The mainstay for the treatment of fetal anaemia is intrauterine blood transfusion (IUT), which is associated with a risk of immunisation to additional antigens, despite the usually small volume of the feto-maternal haemorrhage (FMH) of just a few millilitres. In a cohort of more than 300 women, 25% formed additional antibodies after IUT treatment, and after delivery more than 70% possessed multiple RBC antibodies[1,2]. There is some indication that pregnancy-induced anti-D may persist longer than transfusion-induced D-antibodies[3]. IUT is associated with increased FMH, containing viable HLA-haplo-identical fetal blood cells. It is not known whether post-pregnancy persistence of fetal cells contributes to maintenance of antibody production.

Nowadays most study groups, including ours, report perinatal survival rates in RBC alloimmunization treated with IUT for alloimmune fetal anaemia of above 90%[4-7]. This improved perinatal survival causes a shift in attention towards the short- and long-term outcome in surviving children. To date only a few studies with small patient numbers (range 16 to 69) have reported on the long-term neurodevelopmental outcome[8-14]. Little is known on the association between hydrops fetalis and the severity of fetal anaemia and long-term neurodevelopmental outcome[6].

We started a long-term follow up study of a large cohort to determine 1. Factors involved in the formation of blood group antibodies and the long-term maternal immunological effects after IUT and 2. The incidence of long-term neurodevelopmental impairment (NDI) and associated perinatal risk factors. This study, named the LOTUS study (LOng Term follow-up after intra Uterine transfusionS), is conducted by a consortium of several disciplines involved in fetal transfusions: the Sanquin Blood Supply Foundation and the LUMC: the departments of Obstetrics, Neonatology and ImmunoHematology & Bloodtransfusion.

### Aims

This study is conducted with two separate aims.

#### Part 1: "Long-term maternal immunological outcome"

The first aim is to investigate factors influencing RBC antibody incidence and persistence.

The objectives are:

1. to measure the incidence of RBC antibodies prior to and against RBC and HLA antigens after IUT treatment

2. to investigate whether antibodies present after delivery persisted and whether the fetus or the IUT donor (or both) had provided the offending antigen

3. to investigate whether formation of particular RBC antibodies is associated with maternal Major Histocompatibility Complex (MHC) class II alleles

4. to evaluate whether fetal and/or donor (despite irradiation of RBC) chimerism is associated with persistence of RBC antibodies

5. to study the long-term maternal health effects by a protocolled questionnaire aiming to identify possible maternal immune deviations (e.g. autoimmune diseases) and a screening for the presence of autoimmune antibodies

#### Part 2: "Long-term paediatric neurodevelopmental outcome"

The second aim is to study the long term health and neurodevelopment in children post IUT treatment. The objectives are:

6. to determine the incidence of NDI

7. to investigate the risk factors for developing long-term NDI. Potential risks may include: prematurity, severity of fetal anaemia, presence and severity of fetal hydrops, number of IUT procedures, severe neonatal morbidity (including respiratory distress syndrome, intraventricular haemorrhage ≥ grade 3[15], periventricular leukomalacia ≥ grade 2[16], necrotising enterocolitis ≥ grade 2[17] and/or sepsis) and perinatal asphyxia.

## Methods

### Study population

The Leiden University Medical Center (LUMC) is the single national referral centre in the Netherlands for the management of pregnancies complicated by severe fetal anaemia. All women and their live-born children, treated with IUT for severe fetal alloimmune anaemia from 1987-2008 (20 years) in the LUMC are asked to participate in the study. Women with no live-born children will be excluded. In the period 1987-2008 340 women gave birth to 395 living children.

The LOTUS study started on September 1^st ^2008. To date (January 2010) we included 225 families with 258 children. Seven families refused to participate, 3 families only filled out the questionnaires and 9 families had moved abroad. The lost-to-follow-up rate is currently 16%. We expect an inclusion rate above 80% and to finalise the inclusion period by the end of 2010.

### Procedure

Mothers and children are invited to visit one of our outpatient clinics. After informed consent, in case of children less than 16 years of age provided by parents or care-taker, blood samples are withdrawn from both mothers and children. From young children, instead of a blood sample, a mouth-swap or a saliva sample can be taken. From all women, RBC blood group antigens are known (Rhesus, Kell, Duffy, Kidd and MS), as well as the RBC antibody specificities prior to IUT and after delivery. RBC antigen profile for clinically relevant antigens of fetal and (IUT) donor RBC is completed as much as possible from the databases of Sanquin and the LUMC. Missing RBC and HLA antigens and antibodies will be typed. Physical and neurological examination is performed in all children between 2 to 22 years of age. An evaluation of cognitive and psychomotor development is performed in all children between 2 to 17 years of age.

### Data collection

The following maternal and fetal baseline characteristics are collected from the LUMC Rhesus-database:

- Maternal and fetal baseline characteristics

a. Type of red cell alloimmunization

b. Course and level of Antibody-Dependent Cell-mediated Cytotoxic assay (ADCC) during pregnancy (if applicable)

c. Number of IUT

d. Route of IUT transfusion & estimated FMH

e. Perinatal maternal transfusions

f. Fetal haemoglobin (Hb) concentration before IUT

g. Presence of severe fetal anaemia (>5 SD from reference [Hb] for gestational age)

h. Presence of fetal hydrops, classified as mild or severe

The following neonatal baseline characteristics are extracted from the medical charts:

a. Birth weight, gestational age, gender

b. Haemoglobin concentration at birth

c. Use of exchange transfusions

d. Use of top up red cell transfusion until 3 months of life

e. Neonatal morbidity: kern-icterus, sepsis, respiratory distress syndrome, necrotising enterocolitis[17], retinopathy of prematurity [18], intraventricular haemorrhage[15], periventricular leukomalacia[16], postpartum hydrops fetalis requiring drainage of ascites or pleural effusion, cholestasis, sepsis (defined as a proven infection, a positive blood culture, and clinical signs of infection)

f. Perinatal asphyxia (defined as three of more of the following five criteria: non-reassuring CTG patterns, umbilical cord arterial pH less than 7.10, Apgar score less than 5 at 5 minutes, failure of spontaneous breathing at 5 minutes, onset of multiple organ failure)

### Study outcome

#### Part 1: "Long-term maternal immunological outcome"

The primary outcome is an association between MHC class II alleles and particular RBC antibodies. Most RBC polymorphisms are based on single nucleotide differences. Red cell alloimmunization needs the indirect pathway of antigen stimulation. IgG antibodies can only be formed if T-cell help is signalled to the appropriate B cells. Differential immunogenicity of mismatched antigens is dependent on MHC-restricted potential of antigen presentation, leading to activation of T cells. The frequencies of specific HLA class II antigens will be compared in women with and without specific RBC antibodies with a chi-square or Fisher exact test.

##### Antibodies

All women are tested for the persistence of the various RBC antibodies present after the last IUT and new developed additional antibodies by standard serologic techniques. HLA antibodies are screened by ELISA and specificity is determined by luminometer-based technology. The data on RBC and HLA antigens of children and donors will be completed. RBC antigen profiles of children and donors will be compared with antibody specificities that disappeared or persisted to determine whether the IUT donor and/or the fetus provided the offending antigens. Using MCH typing, we aim to assess MHC restriction of (specific) RBC antibodies.

Microchimerism will be determined in whole blood and, where applicable, within leukocyte subpopulations by Rhesus D and Y chromosome specific RT-PCR.

The presence of fetal chimerism and the nature of the chimeric cells in women with persistent (RBC/HLA) antibodies will be compared to women in whom antibodies are no longer detectable.

##### Maternal health assessment

Mothers are asked to fill out a questionnaire with the emphasis on development of autoimmune disease. Maternal blood samples are screened for a selection of auto-antibodies e.g. relevant for scleroderma.

#### Part 2: "Long-term paediatric neurodevelopmental outcome"

The primary outcome is the composite outcome termed NDI, which is defined as the presence of at least one of the following: abnormal neurological outcome (cerebral palsy), cognitive or psychomotor development score < 2 SD, bilateral blindness, and bilateral deafness requiring amplification. The following potential predictors for NDI will be studied in a univariate logistic regression model: praematurity, severity of fetal anaemia, presence and severity of fetal hydrops, number of IUT procedures, severe neonatal morbidity (including respiratory distress syndrome, intraventricular haemorrhage ≥ grade 3[17], periventricular leukomalacia ≥ grade 2[18], necrotising enterocolitis ≥ grade 2[15] and/or sepsis) and perinatal asphyxia. Multivariable logistic regression model will be used to measure the independent effects of potential predicting factors on outcome.

##### Neurological outcome

In this study the neurological outcome is assessed in all children by a single pediatrician by performing a neurological examination according to Touwen [19] and scored as normal, minor neurological dysfunction, or abnormal. A minor neurological dysfunction is defined as a moderate abnormality of tone, posture, and movement leading to only minor functional impairment or a minor developmental delay. Abnormal development is defined as severe abnormality of tone, posture, and movement leading to functional impairment and/or a delay in motor development. Presence of cerebral palsy (CP) is assessed according to the criteria of the European CP Network and classified as diplegia, hemiplegia, quadriplegia, dyskinetic or mixed [20].

##### Cognitive and psychomotor development

Cognitive and psychomotor development of children aged 2 to 3 years is assessed according to the Dutch version of the Bayley Scales of Infant Development, 2^nd ^edition (BSID-II)[21]. BSID-II scores provide a mental developmental index (MDI) and a psychomotor developmental index (PDI). The MDI and PDI follow a normal distribution curve with a mean score of 100 and a standard deviation of 15. Scores are classified into one of three categories: normal limits (standard scores of ≥ 85); mildly delayed (standard scores of 70-84); and significantly delayed (standard scores of ≤ 70).

Children between 3 and 7 years of age are tested with the Dutch version of the Wechsler Preschool Performance Scale of Intelligence, 3^rd ^edition (WPPSI-III-NL) [22]. Cognitive development in children between 7 and 16 years of age is assessed with the Dutch version of the Wechsler Intelligence Scale for Children, 3^rd ^edition (WISC-III-NL) [23]. Both tests provide a full scale IQ score, a Verbal IQ and a Performance IQ. Scores have a mean of 100 and a SD of 15. Scores of <70 are classified as extremely low, 70 to 79 as borderline, 80 to 89 as low average, 90 to 110 as average, 111 to 119 as high average, 121 to 130 as superior, and ≥ 130 as very superior. All tests are performed by a licensed psychologist.

##### Psychosocial functioning and Health related Quality of life

Psychosocial follow-up is assessed in children between 4 years to 16 years of age using the Dutch version of the Strengths and Difficulties Questionnaire (SDQ) [24]. The SDQ is available as an informant-rated version for children aged 4 years and older (to be completed by parents, teachers or caregivers). The SDQ assesses mental and behavioural difficulties and strengths along the following dimensions: emotional symptoms, conduct problems, hyperactivity/inattention, peer problems and prosocial behaviour. Each scale consists of 5 items, each rated on a 3-point scale (not true, somewhat true and certainly true). Higher scores indicate more problems on the respective difficulties dimension or less appropriate behaviour for the prosocial scale. In addition to dimension-specific scores, the first 4 scales can be summed up to a total difficulties score, with higher scores indicating more problems.

Health related Quality of Life (HRQoL) is assessed in children between 6 years and 22 years of age using the TNO AZL Child Quality Of Life (TACQOL) [25] and the TNO Adult Quality of Life (TAAQOL)[26]. The TACQOL child- and parent form cover 7 eight-item domains of life: physical complaints, motor functioning, autonomy, cognitive functioning, social functioning, positive emotions and negative emotions. The TACQOL assesses the frequency of difficulties experienced in 'the last few weeks' with response categories: never, occasionally or often. If a difficulty is experienced occasionally or often, the emotional reaction to this problem is determined. Items are scored by assigning a value of 4 to the 'never' response, a value of 3 to a 'feeling fine' response, a value of 2 to a 'feeling not so good response, a value of 1 to a 'feeling quite bad' response and a value of 0 to a 'feeling bad' response. The scores in each domain range from 0 to 32. The scales for positive and negative emotions, asking for the frequency of moods on a 3-pointscale (score 0 'never', score 1 'occasionally', score 2 'often'), range from 0 to 16. In all seven domains higher scores indicate better HRQoL.

The TAAQOL covers 12 domains: gross motor functioning, fine motor functioning, pain, sleeping, cognitive functioning, social functioning, daily activities, sexual activity, vitality, happiness, depressive moods, and aggressiveness. First the frequency of occurrence of a specific complaint or limitation during the last month is asked. If such a problem has occurred, the subjective appraisal of this problem is assessed. A score of 1 is given when there is no limitation, a score of 2 when there is a limitation ('a little', 'some' or 'a lot') but when the person is not bothered by this limitation; a score of 3 when there is a limitation and the person experiences this limitation 'a little' negatively; a score of 4 when there is a limitation and the person experiences this limitation 'quite a lot' negatively; and a score of 5 when there is a limitation and the person experiences this limitation 'very much' negatively. Scores of each subscale are normalised to a scale ranging from 0 to 100, with higher scores indicating better HRQoL.

Life-achievement forms based on the POPS-19 study are obtained in children between 8 and 22 years of age. All tests and questionnaires for the parents and children are summarised in table 1.

**Table 1:** An overview of child and proxy questionnaires and tests for paediatric neuromotor, cognitive and psychosocial development in all children treated with IUT for alloimmune fetal anaemia.

Age	Child Questionnaire	Proxy Questionnaire	Life Achievement	Cognitive Development
2				BSID-III-NL
				
3				
				
4				
5			Proxy questionnaire	WPPSI-III-NL
6				
				
7				
				
8				
9				
10				
11				
12	TACQOL	SDQ/		
13		TACQOL		
14				
15				
				
16				WISC-III-NL
17			Child questionnaire	
18				
19				
20	TAAQOL			
21				
22				

*TACQOL *TNO AZL Child Quality of Life, *TAAQOL *TNO AZL Adult Quality of Life, *SDQ *Strengths and Difficulties Questionnaire, *BSID-II-NL *Bailey Scales of Infant Development 2^nd ^edition, *WPPSI-III-NL *Wechsler Pre-school Performance Scale of Intelligence 3^rd ^edition, *WISC-III-NL *Wechsler Intelligence Scale for Children 2^nd ^edition.

### Ethical approval

The LOTUS study was approved by the ethics committee of the Leiden University Medical Centre on June 1^st ^2008 (P08.080).

## Discussion

### Part 1: "Long-term maternal immunological outcome"

Pregnant women with Rh-D alloimmunization resulting in haemolytic disease of the fetus, receive a median of 3 (range 1-8) intrauterine transfusions (IUT). The treatment is associated with a high risk on additional maternal RBC antibody production and the immunisation rate exceeds that of any poly-transfused patient group [1,2]. The mechanism of this high immunisation rate is still unknown and the aim of this study is to unravel several factors that may contribute to this phenomenon.

MHC-restriction in more or less efficient presentation of RBC antigens may play a role. According to the study by Noizat-Pirenne et al [27,28], all patients with anti-Fy^a ^had a specific HLA DRB1 phenotype, compared to 19% in the ethnic control population. Less specific relationships were found for the Jk^a ^[28,29] and K [27] antigen.

In uncomplicated pregnancy, approximately 15-30% of single- or multiparous women form antibodies directed against inherited paternal HLA antigens of the fetus [30-32]. The current study investigates HLA alloimmunization in pregnancies complicated by maternal RBC immunisation, in order to explore whether high antibody responder ship and persistence of antibodies is associated with RBC immunisation and/or fetal microchimerism [33]. Several studies have described a relationship between high levels of microchimerism and the increased risk on auto-immune diseases, e.g. systemic sclerosis [34-36]. We will investigate whether these mothers are at an increased risk for the development of auto-immune diseases.

The results of this study may lead to a change in transfusion policy, in particular future avoidance of certain mismatches.

### Part 2: "Long-term paediatric neurodevelopmental outcome"

Only a few small studies have focused on the paediatric long-term neurodevelopmental outcome after IUT. Most follow-up studies showed that despite the severity of fetal haemolytic disease, developmental outcome for children treated with IUT is usually normal[4-7,10]. Doyle et al. reported on the sensorineural outcome at 2 years of age in 38 survivors of IUTs. The majority of these infants (92%) showed no sensorineural disability at 2 years of age [8]. Hudon et al. studied the neurodevelopmental outcome in 40 infants with HDFN treated with IUT. All infants showed normal developmental outcome at the age of 62 months [11]. Grab et al. described 35 infants treated with IUTs for severe erythroblastosis. At 6 years of age no moderate or severe neurological impairment was observed [12]. Harper et al. evaluated long-term outcome in 18 hydropic fetuses treated with IUT. Death or major neurological morbidity occurred in 22% of the fetuses and 12% of the survivors had major neurological sequelae[13]. The largest follow-up study to date was performed by our group more than a decade ago and included 69 infants with HDFN. We found that the neurodevelopmental outcome for children with HDFN treated with IUTs compared favourably with a group of high-risk, very low birth weight infants (10% versus 18%, respectively), but less favourably with a healthy control group (10% versus 6%, respectively) [10]. The main limitation of all studies is the small number of patients included (from 16 to a maximum number of 69 children). Moreover, cognitive development and quality of life assessment were often not reported.

Importantly, although fetal hydrops was found to be associated with increased mortality, not much is known about the association between the severity of fetal anaemia or other risk factors and long-term neurodevelopmental outcome [7].

This study will address long term outcome in a large number of children (>330) and will enable us to determine the association between several, potentially clinically relevant risk factors and long-term outcome. The LOTUS study will provide clinicians and parents better insights in the long-term outcome of children with RBC alloimmunization treated with IUTs, and may improve the quality of antenatal counselling on long-term neurodevelopmental outcome.

## List of abbreviations

ADCC: Antibody-Dependent Cell-mediated Cytotoxicity assay; BSID: Bayley Scales of Infant Development; FMH: Fetomaternal haemorrhage; HDFN: Haemolytic Disease of the Fetus and Newborn; HLA: Human Leucocyte Antigen; IUT: IntraUterine Transfusion; MDI: Mental Development Index; MHC: Major Histocompatibility Complex; NDI: NeuroDevelopmental Impairment; PDI: Psychomotor Development Index; RBC: Red Blood Cell; RT-PCR: Real Time Polymerase Chain Reaction; SDQ: Strenghts and Difficulties Questionnaire; TAAQOL: TNO AZL Adolescent Quality of Life; TACQOL: TNO AZL Child Quality of Life; WISC-III: Wechsler Intelligence Scale for Children-3^rd ^edition; WPPSI-III: Wechsler Preschool Performance Scale of Intelligence-3^rd ^edition

## Competing interests

The authors declare that they have no competing interests.

## Authors' contributions

VS, HS, IK, DO, ID, EL and AB were involved in the conception and design of the study. EV, IL, HS and EL have drafted this manuscript. All authors are members of the LOTUS study group. All authors edited the manuscript and read and approved the final draft.

## Pre-publication history

The pre-publication history for this paper can be accessed here:

http://www.biomedcentral.com/1471-2393/10/77/prepub
